# Logistics of clinical xenotransplantation: from donor pig production to global organ distribution

**DOI:** 10.3389/ti.2026.17326

**Published:** 2026-10-15

**Authors:** Konrad Fischer, Joachim Denner, Ralf Reinhard Tönjes, Philipp Felgendreff, Axel Rahmel

**Affiliations:** 1 Walter Brendel Center of Experimental Medicine, Institute of Surgical Research, University Hospital, LMU Munich, Munich, Germany; 2 Institute of Virology, Free University Berlin, Berlin, Germany; 3 Paul-Ehrlich-Institut, Federal Institute for Vaccines and Biomedicines, Langen, Germany; 4 Department of General, Visceral and Transplant Surgery, Hannover Medical School, Hannover, Germany; 5 German Organ Transplantation Foundation, Frankfurt, Germany

**Keywords:** biosafety, logistics, machine perfusion, organ preservation, xenotransplantation

## Abstract

The shortage of transplantable organs remains one of the most significant unmet challenges in modern medicine. Despite advances in donor management, organ preservation, surgical techniques, and immunosuppression, organ demand continues to exceed supply worldwide. Xenotransplantation using genetically engineered porcine organs has emerged as a promising strategy to address this gap. Recent clinical pig-to-human heart and kidney transplantations have demonstrated the feasibility of clinical xenotransplantation and shifted attention from immunological barriers toward practical implementation. This review examines the logistical requirements for clinical xenotransplantation, with particular emphasis on donor herd management, biosafety, organ preservation technologies, and future distribution networks. The integration of donor production with advanced machine perfusion technologies may ultimately transform transplantation medicine from a donor-dependent emergency service into a predictable and scalable therapeutic platform.

## Introduction

The global shortage of transplantable organs continues to limit the effectiveness of transplantation medicine. Although transplantation remains the preferred treatment for end-stage organ failure, organ availability falls far short of clinical demand (see Table 1). In the United States, more than 103,000 patients are currently registered on organ waiting lists, while approximately 17 patients die each day before receiving a suitable graft. Similar challenges exist in Europe, where approximately 60,000 patients await transplantation despite more than 34,000 transplant procedures being performed annually [1, 2].

**TABLE 1:** Organ demand and transplant activity in major global regions (2024–2025).

Parameter	Europe	United States	China	India
Population	∼449 million	∼340 million	∼1.41 billion	∼1.43 billion
Patients on waiting lists	∼60,000	>103,000	Not fully reported	Not fully reported
Annual organ transplants	∼34,000	∼48,000–49,000	∼20,000–25,000*	∼18,000–20,000*
Annual kidney transplants	∼22,000–25,000	∼27,000	∼13,000–15,000	∼13,500
Annual liver transplants	∼8,000–9,000	∼10,000	∼6,000–8,000	∼4,000–5,000
Annual heart transplants	∼2,000–2,500	∼4,500	∼500–700	∼300–500
Annual lung transplants	∼2,000	∼3,000	<300	<200
Donation rate (pmp)	20–35	∼44.5	∼3.9	∼0.8–1.0

Reliable national waiting-list statistics are available for Europe and the United States but remain inconsistently reported in several Asian countries. Therefore, estimates for China and India are based on transplantation activity, end-stage organ failure prevalence, and published assessments of unmet transplant need rather than official waiting-list registries [1–7]. *Approximate values from national reports and reviews.

The shortage is even more pronounced in emerging economies. China performs more than 20,000 organ transplants annually but reports deceased donor rates below 4 donors per million population, substantially lower than rates observed in North America and Western Europe. In India, approximately 100,000 patients require kidney transplantation each year, yet only about 13,500 procedures are performed, leaving the majority of patients without access to definitive treatment [3].

Against this background, xenotransplantation has emerged as one of the most promising approaches to expand organ availability. Pigs are considered ideal donor animals because of their physiological similarities to humans, short gestation periods, large litter sizes, and amenability to genetic engineering. Following decades of experimental work, recent clinical milestones have accelerated the translation of xenotransplantation into clinical practice. In 2022, genetically modified pig kidneys demonstrated immediate function following transplantation into brain-dead human recipients [8, 9], while the first life-supporting pig-to-human heart transplantation provided proof of principle for cardiac xenotransplantation [10, 11]. More recently, successful kidney xenotransplantations in living recipients have demonstrated sustained graft function and survival, representing a major advance toward clinical implementation [12, 13].

Historically, xenotransplantation research focused predominantly on overcoming immunological incompatibilities. However, recent clinical progress has highlighted a second challenge that may determine the scalability of the field: logistics. Unlike conventional transplantation, xenotransplantation enables planned organ production and therefore requires an integrated supply chain encompassing donor animal breeding, pathogen surveillance, organ procurement, preservation, transportation, allocation, and long-term recipient monitoring.

In many respects, future xenotransplantation systems may resemble biopharmaceutical manufacturing networks more closely than traditional organ donation systems. This review focuses on the logistical infrastructure required for routine clinical xenotransplantation, emphasizing donor production, biosafety, organ preservation, and future distribution models.

## Donor pig production: from genetic engineering to clinical organ supply

The foundation of clinical xenotransplantation is the availability of genetically engineered donor pigs capable of providing safe and functional organs. Recent advances in genome editing have transformed donor pigs from immunologically incompatible animals into increasingly human-compatible biological platforms [14–16].

Most contemporary donor pigs carry multiple genetic modifications. The elimination of the major xenoantigens αGal, Neu5Gc, and Sd(a) through knockout of the GGTA1, CMAH, and B4GALNT2 genes substantially reduce rejection responses. Additional human transgenes further improve compatibility with human immune and coagulation systems [17]. The donor pig used in the first clinical heart xenotransplantation carried 10 genetic modifications, illustrating the increasing complexity of modern donor animals [10].

From a logistical perspective, genetic engineering introduces significant production challenges. Unlike conventional livestock breeding, donor herds must maintain highly specific genotypes while simultaneously meeting strict microbiological standards. Production therefore relies on advanced reproductive technologies including somatic cell nuclear transfer, embryo transfer, artificial insemination, and cryopreservation. A central question for future clinical implementation concerns donor herd size. Europe currently performs approximately 22,000–25,000 kidney transplants annually. Assuming that each donor pig provides two transplantable kidneys, a theoretical minimum of 10,000–12,500 donor pigs would be required to replace current kidney transplantation activity. However, donor exclusion, organ discard, transportation losses, and reserve capacity substantially increase actual requirements. Realistic estimates suggest that approximately 15,000–20,000 donor pigs annually may be necessary to support kidney xenotransplantation programs across Europe (see Tables 2, 3). Similar calculations suggest requirements of approximately 18,000–20,000 donor pigs annually in the United States [21].

**TABLE 2:** Estimated annual donor pig requirements.

Region	Kidney allo-transplants currently performed	Estimated annual need	Estimated pigs required (kidneys only)
Europe	25,000	30,000–40,000	15,000–20,000
USA	27,000	35,000–40,000	18,000–20,000
China	15,000	150,000–300,000	75,000–150,000
India	13,500	100,000	50,000–75,000

Donor pig requirements were calculated assuming two transplantable kidneys per donor pig and adjusted for expected donor exclusion, organ discard, and reserve capacity [3, 7, 18, 19].

**TABLE 3:** Regional potential demand for xenotransplantation.

Region	Current transplant capacity	Estimated unmet demand	Potential xenotransplant market
Europe	High	Moderate	High
USA	High	High	High
China	Moderate	Very high	Extremely high
India	Low-moderate	Extremely high	Extremely high
Japan	Moderate	High	High

Potential demand is estimated according to recent publications [1, 3, 5, 20].

Demand may be substantially greater in Asia. Given the large unmet transplantation needs in China and India, future xenotransplantation programs could require tens of thousands of additional donor animals annually. Consequently, donor production facilities must be designed for industrial-scale operation rather than experimental research.

Importantly, the effective organ yield per donor pig may substantially reduce overall donor requirements. In addition to two kidneys, a single donor animal may provide a heart, lungs, liver, corneas, pancreatic islets, vascular grafts, and heart valves. Coordinated multi-organ allocation could therefore maximize utilization and reduce the number of animals required to support national transplantation programs (see Table 4). Modern commercial pig breeding systems typically achieve 25–30 weaned piglets per sow per year, while highly optimized specific- or designated pathogen-free (DPF) systems may achieve slightly lower production due to stricter biosecurity requirements. Donor herd requirements should be regarded as a function of reproductive performance and sequential losses during production rather than simply the theoretical number of piglets produced per sow. Commercial breeding data indicate approximately 2.4 litters per sow per year and pre-weaning survival rates of approximately 85%–90%. However, these values cannot necessarily be extrapolated directly to high-containment DPF production. Experience from DPF source-animal facilities demonstrates that additional losses may occur during herd establishment and maintenance, including substantial neonatal mortality and exclusion associated with microbiological events. Accordingly, a simplified annual donor-output model can be expressed as Y = L × P × S_p_ × S_n_ × QDPF × QG, where *L* is litters per sow per year, *P* is live-born piglets per litter, *S*_*p*_ and *S*_*n*_ represent pre-weaning and subsequent production survival, respectively, *QDPF* is the proportion maintaining DPF qualification, and *QG* is the proportion fulfilling genetic and organ-specific donor criteria. The required number of productive sows is therefore Nsow = D/Y, where *D* represents the targeted annual number of clinically eligible donor pigs. Breeding-herd turnover should additionally be considered because commercial sow replacement rates commonly approach 40%–50% annually. Because robust large-scale performance data from clinical-grade DPF xenotransplantation facilities remain unavailable, these calculations should be interpreted as scenario-based estimates rather than fixed predictions [22–25]. Assuming an average output of 25 donor-qualified piglets per breeding sow annually and a donor qualification rate of approximately 70%–80%, a facility producing 5,000 clinically eligible donor pigs per year would require approximately 250–300 breeding sows and an overall herd size of 3,000–5,000 animals including boars, replacement gilts, nursery pigs, and production animals.

**TABLE 4:** Potential number of donor pigs and breeding facilities required for national xenotransplantation programs.

Region	Annual donor pigs required	Estimated breeding sows	Approximate total herd size	Number of facilities
Germany	5,000–6,000	250–350	3,000–5,000	1
France	4,000–5,000	200–250	2,500–3,000	1
UK	3,500–4,500	175–225	2,000–3,000	1
Europe	15,000–20,000	800–1,200	10,000–15,000	3–5
United States	18,000–25,000	900–1,200	12,000–15,000	3–5
China	75,000–180,000	4,000–9,000	50,000–100,000	10–20
India	50,000–75,000	2,500–4,000	30,000–60,000	5–10

Assuming xenotransplantation eventually replaces most kidney waiting-list demand. Transplantation of up to 2 kidneys per pig [1–5, 19].

For comparison, replacing the current annual kidney transplant activity in Germany alone (approximately 5,000–6,000 donor pigs annually) would require one dedicated large-scale donor production facility (see Table 4). Implementation in Europe or the USA would likely necessitate 3 to 5 production centers (Figure 1). In contrast, meeting only a fraction of the projected kidney transplant demand in China could require donor production capacities exceeding 100,000 animals annually, necessitating multiple industrial-scale facilities distributed across the country.

**FIGURE 1:**
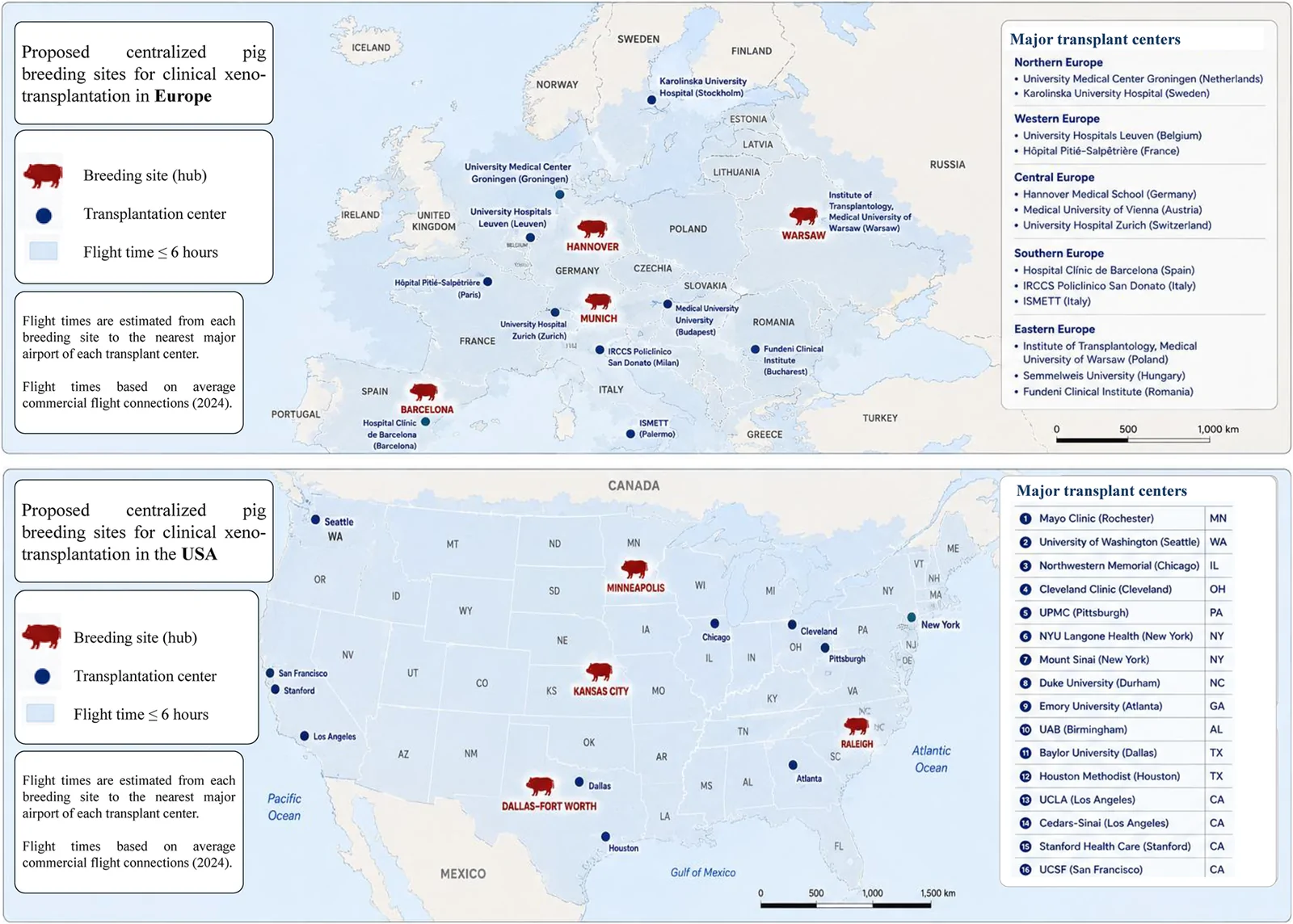
Hypothetical locations of donor pig breeding facilities for future clinical xenotransplantation networks in Europe and the USA. The selected locations allow access to major transplantation centers within approximately 6 h of flight time (currently shortest possible cold ischemia time) using current transportation and organ preservation technologies. Site selection considered geographic coverage, transportation logistics, biomedical infrastructure, air-cargo capacity, transplantation activity, and regional resilience. In Europe, Barcelona, Munich, Hannover, and Warsaw represent potential breeding facility locations serving Southern, Central, Northern, and Eastern Europe, respectively. In the United States, facilities located near Minneapolis, Kansas City, Raleigh-Durham, and Dallas-Fort Worth could provide nationwide coverage. Continued advances in machine perfusion and organ preservation may enable further centralization of donor animal production and reduce the number of facilities required.

## Biosafety and pathogen surveillance

While immunological barriers have received considerable attention, biosafety remains one of the defining challenges of clinical xenotransplantation. Because transplantation occurs across species barriers, concerns extend beyond graft survival to include potential zoonotic transmission and public health implications [26]. To minimize infectious risks, donor pigs are maintained within specific pathogen-free (SPF) or designated pathogen-free (DPF) breeding facilities. SPF refers to animals maintained free of a predefined set of pathogens under controlled biosecurity conditions. In xenotransplantation, DPF status is more specifically based on the exclusion of pathogens considered relevant to xenograft safety and potential zoonotic transmission. Thus, the DPF pathogen panel is defined according to the intended clinical use and corresponding risk assessment rather than representing a universally fixed list of microorganisms. DPF facilities represent the currently highest-possible standard and operate under conditions resembling high-containment biomedical production environments. Donor animals are housed in positive-pressure barrier units equipped with HEPA-filtered ventilation, sterilized feed and water systems, restricted personnel access, and continuous environmental monitoring [23].

Xenotransplantation logistics must also include reverse logistics and biological risk management. In addition to the forward transport of donor organs, biosecure production networks require predefined procedures for the isolation of animals or units following suspected pathogen exposure, controlled movement restrictions during outbreaks, and the safe removal and disposal of culled or retired donor animals. Likewise, biological samples, contaminated materials, and medical waste generated during donor production, procurement, transport, and transplantation must be handled through validated containment, decontamination, and disposal pathways. Such reverse-logistics processes are essential to prevent pathogen dissemination between breeding facilities, procurement units, transplant centers, and the surrounding environment and should therefore be considered an integral component of the xenotransplantation supply chain.

Pathogen surveillance programs target a broad range of viral, bacterial, and parasitic agents. Particular attention is directed toward porcine cytomegalovirus, which is a porcine roseolovirus, (PCMV/PRV), porcine reproductive and respiratory syndrome virus, hepatitis E virus, circoviruses, influenza viruses, and porcine endogenous retroviruses (PERVs) [27–30]. The importance of rigorous screening became evident following the first clinical pig-heart transplantation, during which PCMV/PRV was detected in the donor organ and has contributed to graft dysfunction [31]. Preclinical xenotransplantation studies in non-human primates have shown that PCMV/PRV markedly reduces xenotransplant survival [32, 33]. For an effective screening, donor pigs should be screened for PCMV/PRV and other relevant viruses using both PCR-based and immunological assays [34]. Such testing requires specialized facilities, ideally located near the breeding or transplantation centers [35].

PERVs present a unique challenge because they are integrated into the porcine genome and cannot be eliminated through conventional biosecurity measures. Although no clinical transmission has been documented, concerns regarding long-term zoonotic adaptation have stimulated extensive research [36, 37]. Recent CRISPR/Cas9 approaches have successfully inactivated multiple PERV sequences within donor pigs, potentially eliminating this risk [38].

Biosafety requirements extend beyond donor production. While lifelong immunosuppressive therapy is required to maintain graft function, current regulatory frameworks additionally recommend lifelong recipient monitoring, donor tissue archiving, and centralized surveillance programs. Future xenotransplant recipients will likely undergo regular virological screening and participate in national registries designed to detect emerging infectious risks. Consequently, biosurveillance must be viewed not as a separate regulatory requirement but as an integral component of xenotransplantation logistics. Considering that viruses such as HIV-1, human cytomegalovirus (HCMV), Epstein-Barr virus (EBV), and even rabies virus have been transmitted through allotransplantation, xenotransplantation may ultimately prove to be safer than allotransplantation with respect to infectious disease transmission.

## Organ procurement, preservation, and distribution: the core xenotransplantation supply chain

The transition from experimental xenotransplantation to routine clinical application will depend largely on the establishment of reliable procurement, preservation, and distribution systems. While donor pig production provides the foundation for organ availability, the ability to safely recover, preserve, transport, and allocate organs will ultimately determine whether xenotransplantation can be implemented on a large scale. Unlike conventional decentralized allotransplantation, where donor availability is unpredictable and transplantation frequently occurs under emergency conditions, xenotransplantation offers the possibility of a planned and highly coordinated supply chain.

## Planned organ procurement

One of the most important logistical advantages of xenotransplantation is the ability to schedule organ procurement. In conventional transplantation, organs become available only after donor death and must be allocated rapidly under considerable time pressure. Individual procurement teams are mobilized at short notice, transportation is frequently organized within hours, and recipient preparation often occurs concurrently with organ recovery.

In contrast, xenotransplantation enables elective procurement. Donor animals are maintained under controlled conditions, allowing transplantation procedures to be scheduled in advance. This predictability offers several advantages. Recipients can undergo comprehensive preoperative evaluation, immunosuppressive protocols can be optimized before transplantation, and operating rooms, surgical teams, and intensive care resources can be allocated efficiently. Furthermore, organ procurement can be performed during normal working hours, reducing the logistical burden associated with emergency transplantation procedures.

Before procurement, donor pigs undergo final qualification procedures including genetic verification, microbiological screening, hematological analysis, and organ-specific functional assessment. Because transplantation candidates may already have been selected before organ retrieval, a coordinated donor-recipient matching process becomes possible. Unlike allotransplantation, where HLA compatibility plays a central role, xenotransplant allocation is likely to focus primarily on organ size, blood group compatibility, recipient risk factors, and logistical considerations such as transport distance and preservation time.

A major advantage of xenotransplantation is the possibility of coordinated multi-organ and tissue recovery from a single donor animal. Procurement procedures must therefore be carefully organized to maximize donor utilization while minimizing ischemic injury and ensuring appropriate allocation of the recovered grafts. Future donor facilities will likely contain dedicated procurement suites adjacent to breeding units, enabling rapid transition from donor qualification to organ recovery.

## Machine perfusion: a key enabling technology

Although donor availability represents the primary advantage of xenotransplantation, organ preservation remains a critical challenge and an opportunity for systemic improvement. Traditional static cold storage has served as the standard preservation method for decades but imposes substantial limitations on transportation and allocation. Ischemia-reperfusion injury remains a major contributor to graft dysfunction and organ discard. Besides gene-editing approaches to limit graft damage directly in donor pigs [39], machine perfusion has emerged as one of the most important technological developments in modern transplantation and may become central to future xenotransplantation logistics. By maintaining organ circulation *ex vivo*, machine perfusion can extend preservation times, permit functional assessment, allow continuous monitoring of the graft, and potentially reduce ischemic injury.

Hypothermic machine perfusion (HMP) continuously circulates preservation solution through the organ at low temperatures. When oxygen is actively supplied during hypothermic perfusion, the approach is commonly referred to as hypothermic oxygenated perfusion (HOPE). HOPE combines reduced metabolic demand at low temperature with controlled oxygen delivery, thereby supporting mitochondrial recovery and reducing metabolic injury before reperfusion. Numerous clinical studies in kidney transplantation have demonstrated reductions in delayed graft function and improved graft outcomes compared with static cold storage [40].

Normothermic machine perfusion (NMP) represents an even more transformative technology. Under normothermic conditions, organs remain metabolically active and can be continuously monitored and critically evaluated during the entire preservation period up to the implantation procedure. Parameters such as vascular resistance, oxygen consumption, urine production, bile secretion, and organ-specific function can be monitored in real time. Consequently, NMP transforms preservation from a passive storage process into an active period of organ assessment and optimization [41].

Experimental studies have demonstrated successful preservation of genetically modified porcine kidneys, livers, and lungs using clinically approved perfusion systems [42]. These findings suggest that future xenotransplant programs may depend heavily on perfusion technologies to support large-scale distribution networks.

Importantly, machine perfusion may significantly expand geographical allocation boundaries. Current preservation windows for conventionally stored organs remain limited. Hearts typically tolerate only 4–6 h of cold ischemia, lungs approximately 6–8 h, and livers approximately 8–12 h. Kidneys are more resilient and may remain viable for 24–36 h under static cold storage. Machine perfusion has the potential to extend these windows substantially, with preservation periods exceeding 24 h already reported for several organ types [43, 44].

Extended preservation times fundamentally alter transplantation logistics by reducing the urgency of transportation and permitting broader organ allocation. In practical terms, machine perfusion may allow organs produced in centralized donor facilities to be distributed across entire countries or even continents.

## Transportation and distribution models

The implementation of xenotransplantation requires careful consideration of organ transportation strategies. Because donor pigs are likely to be maintained in a limited number of highly specialized facilities, organs must be transported efficiently to recipient hospitals. This introduces a challenge that differs substantially from current transplantation systems, where donor organs originate from geographically dispersed hospitals. Several organizational models have been proposed.

In a centralized system, donor pigs are produced within one or several large breeding facilities that supply transplant centers across an entire country or region. Centralized production offers significant advantages, including standardized quality control, centralized expertise, efficient pathogen surveillance, and economies of scale. However, centralized systems also create vulnerabilities. Transportation becomes increasingly important, and disruptions affecting a major production facility could have widespread consequences. Furthermore, longer transport distances may increase logistical complexity and preservation requirements.

A decentralized approach involves multiple smaller donor facilities located near transplantation centers. Such systems reduce transportation distances and may improve resilience against local disruptions. However, they require duplication of infrastructure, personnel, and surveillance systems, substantially increasing costs. Maintaining consistent quality standards across multiple facilities may also prove challenging.

A hybrid regional hub-and-spoke model may represent the most realistic solution, particularly in Europe. In this framework, several strategically located donor facilities supply regional perfusion hubs, which in turn distribute organs to transplantation centers. Such a system combines the quality-control advantages of centralized production with the flexibility of regional distribution.

The concept of collaborative perfusion hubs has gained increasing attention in recent years. Stoerzer and colleagues proposed this model in which donor organs are transported from production facilities to specialized perfusion centers for quality assessment, functional testing, and optimization before redistribution to recipient hospitals [42].

Europe is particularly well suited to a regional hub model because of its established cross-border transplantation infrastructure. Existing organizations such as Eurotransplant already coordinate organ allocation among multiple countries and could potentially provide a framework for future xenotransplantation networks. The implementation of xenotransplantation in Asia will face logistical challenges that differ from those in Europe and North America. In countries such as China and India, large geographical distances, pronounced regional differences in healthcare infrastructure, and variable access to reliable cold-chain and air-cargo networks may complicate the distribution of donor organs from centralized production facilities. These challenges may favor a more decentralized or regionally clustered production model, supported by strategically located procurement and perfusion hubs. In addition, heterogeneous national regulatory frameworks, requirements for source-animal production, biosafety surveillance, and cross-regional transport of biological materials may further influence implementation. Thus, although the potential clinical demand for xenotransplantation in Asia is substantial, the development of scalable regional infrastructure will be essential before this demand can be translated into routine clinical access.

## Global perspectives

Current xenotransplantation research is concentrated primarily in North America, Europe, and China. However, the future demand for xenotransplantation is likely to be greatest in Asia. China’s large population and relatively low deceased-donor rate create a substantial unmet need for transplantable organs [4, 45]. Similarly, India performs only a fraction of the kidney transplants required to meet clinical demand [46].

These disparities suggest that future donor production facilities in Asia may need to operate at a scale considerably larger than those currently envisioned in Europe or North America. Consequently, logistics systems developed for xenotransplantation must be scalable and adaptable to diverse healthcare environments.

However, the potential demand for xenotransplantation will not be determined by medical need alone, but also by societal and individual acceptance. Recent surveys from China illustrate this complexity. A study by Wen et al., revealed that although most participants had heard of xenotransplantation, only 4.9% considered themselves very familiar with the concept, and only 36.7% stated that they would definitely accept a xenotransplant in a life-saving situation [47]. Acceptance was influenced by familiarity, sociodemographic factors, and perceived religious conflicts, while concerns focused particularly on safety, postoperative outcomes, and costs. Thus, estimates based solely on unmet transplantation need may overestimate actual clinical demand, and regional implementation strategies will also need to consider public perception, cultural factors, and willingness to accept xenogeneic organs.

Therefore, the integration of donor production, machine perfusion, transportation, and allocation systems represents one of the most important challenges facing clinical xenotransplantation. Success will depend not only on biological compatibility but also on the development of efficient and resilient supply chains capable of delivering organs safely and predictably to recipients.

## Regulatory framework

The successful clinical implementation of xenotransplantation depends not only on scientific and technical advances but also on the establishment of robust regulatory frameworks. Unlike conventional allotransplantation, xenotransplantation intersects with transplantation medicine, infectious disease control, biotechnology regulation, animal welfare, and public health. Consequently, regulatory oversight extends far beyond graft function and patient outcomes.

In the United States, xenotransplantation is regulated primarily by the U.S. Food and Drug Administration (FDA). Specific recommendations are provided in the FDA Guidance for Industry “Source Animal, Product, Preclinical, and Clinical Issues Concerning the Use of Xenotransplantation Products in Humans”*,* which addresses donor animal qualification, pathogen surveillance, product testing, preclinical evaluation, clinical protocols, and long-term recipient monitoring [48].

In Europe, regulatory oversight is distributed among several institutions. The European Medicines Agency (EMA) and EU national competent authorities share responsibility for xenotransplantation-related activities. The European Directorate for the Quality of Medicines and Healthcare (EDQM) at the Council of Europe (CoE) currently compiles information on xenotransplantation activities, research, legislation and regulation in European countries [49]. Existing regulations governing tissues and cells, advanced therapy medicinal products, and transplantation provide a partial framework, although dedicated xenotransplantation-specific regulations remain limited. The CoE and EDQM have repeatedly emphasized traceability, biosafety, and recipient surveillance as central components of future clinical programs.

In the United States, one of the most distinctive regulatory requirements is long-term recipient monitoring. FDA guidance recommends lifelong surveillance of xenotransplant recipients, including continued clinical and laboratory monitoring and archiving of biological specimens. In Europe, Council of Europe recommendations similarly emphasize long-term traceability and monitoring of recipients to identify potential xenogeneic infections and other public-health risks.Because xenotransplantation introduces the possibility of cross-species pathogen transmission, recipients are expected to undergo lifelong surveillance.

Such programs may include periodic blood sampling, microbiological testing, donor tissue archiving, and participation in national or international registries [27]. Long-term clinical follow-up itself does not fundamentally distinguish xenotransplantation from conventional solid-organ transplantation, as both require lifelong immunosuppression and continuous medical supervision. However, xenotransplantation introduces additional requirements related to the surveillance of potential xenogeneic infections, long-term sample archiving, and donor–recipient traceability, thereby adding specific logistical and regulatory components to routine post-transplant care [50].

Traceability represents another essential component. Every xenograft must be linked to the donor animal, breeding facility, genetic background, microbiological screening results, procurement procedures, and recipient outcomes. This level of documentation resembles pharmaceutical manufacturing more closely than conventional transplantation practice and reinforces the concept of xenotransplantation as an integrated biological production system.

Although regulatory requirements may initially increase costs and complexity, harmonization between regulatory agencies will be critical for the future development of international xenotransplantation networks. In particular, Europe may benefit from coordinated standards that facilitate organ exchange among countries while maintaining consistent safety requirements.

## Economic considerations

The economic viability of xenotransplantation remains an important but incompletely understood aspect of future implementation. Xenotransplantation makes it possible to substantially increase the number of transplants, which are expected to require more intensive postoperative care - at least initially - than transplants using human donor organs. This will place significant demands on transplant centers in terms of personnel and infrastructure. Establishing donor breeding facilities, maintaining pathogen-free herds, using novel detection methods for potential pathogens that need to be established and validated, implementing surveillance systems, and deploying machine perfusion technologies require substantial investments. In addition, the necessary staffing and infrastructure must be put in place at transplant centers. Consequently, the initial costs of xenotransplantation are expected to exceed those of conventional transplantation.

An important unresolved component is the cost of the genetically engineered donor organ itself. Cooper previously emphasized that this cost is difficult to estimate because it must incorporate genetic engineering, breeding and maintenance under biosecure conditions, extensive pathogen screening, dedicated infrastructure, and the commercial costs associated with establishing and maintaining donor-pig production [51]. More recent analyses similarly identify the acquisition cost of the gene-edited pig organ as a major uncertainty in assessing the overall economics of clinical xenotransplantation. Thus, robust cost estimates will likely only become possible once donor production and transplantation are performed at larger clinical scale [52]. Construction costs for large-scale donor facilities and ongoing operational expenses including veterinary care, microbiological monitoring, environmental control systems, and specialized personnel will be economically challenging [23].

Machine perfusion technologies also increase procedural costs. However, evidence from conventional transplantation suggests that these technologies may ultimately improve cost-effectiveness by reducing organ discard rates, minimizing ischemia-reperfusion injury, and improving graft survival [43]. If machine perfusion enables broader organ utilization and supports large-scale distribution networks, the additional costs may be offset by improved clinical outcomes.

Economic comparison with current therapies is particularly important for kidney transplantation. Dialysis represents a substantial and recurring healthcare expenditure, whereas kidney transplantation is associated with higher initial costs followed by markedly lower annual treatment costs. Annual costs for dialysis frequently exceed €60,000–100,000 per patient in Europe and approximately $90,000 per patient in the United States. Over a period of several years, cumulative dialysis expenditures often exceed the anticipated costs of transplantation. Registry-based data from Sweden showed that transplantation became substantially less costly than continued dialysis from the second year after transplantation onward. More recently, an analysis across 14 EU countries found kidney transplantation to be economically favorable in all countries examined, with a mean estimated break-even time of approximately 3.1 years. Thus, despite the high initial costs of transplantation, cumulative dialysis expenditures may exceed transplantation costs within only a few years, making successful long-term transplantation economically attractive [53, 54].

The economic implications become even more significant when considering countries with large unmet transplantation needs. In China and India, where millions of patients suffer from chronic kidney disease and transplantation capacity remains limited, scalable xenotransplantation systems could substantially reduce long-term healthcare expenditures associated with dialysis and chronic organ failure.

Importantly, the economics of xenotransplantation will depend heavily on scale. Initial procedures will likely be costly because of limited production capacity and extensive regulatory requirements. However, expansion and standardization of donor production may create economies of scale and reduce the cost per transplantable organ. Comparable developments have occurred in biopharmaceutical manufacturing, particularly for monoclonal antibodies and recombinant proteins, where increased production scale, higher process yields, standardized manufacturing platforms, and process intensification have contributed to reductions in manufacturing cost of goods.

Thus, while xenotransplantation will initially represent a high-cost intervention, long-term economic analyses may ultimately demonstrate favorable cost-effectiveness, particularly for kidney transplantation and chronic organ failure management.

## Conclusion

Recent clinical advances have transformed xenotransplantation from an experimental concept into a realistic therapeutic option for organ replacement. While overcoming immunological barriers remains important, the future success of the field will depend equally on the establishment of robust logistical infrastructures capable of supporting large-scale clinical application.

Clinical xenotransplantation requires a comprehensive supply chain extending from donor pig production to long-term recipient follow-up. This chain encompasses genetic engineering, pathogen-free breeding facilities, biosafety surveillance, organ procurement, machine perfusion, transportation, regulatory oversight, and recipient monitoring. Together, these components form an integrated system that differs fundamentally from conventional allotransplantation.

Machine perfusion technologies are expected to play a central role. Beyond extending preservation times, future perfusion systems may allow organ repair, immunological conditioning, and real-time quality assessment. The concept of regional perfusion hubs represents an important step toward the creation of continent-wide organ distribution systems.

Beyond machine perfusion, whole-organ cryopreservation may represent a future strategy for long-term xenograft banking. Advances in vitrification and nanowarming have enabled cryogenic storage of rat kidneys for up to 100 days followed by successful transplantation and restoration of life-sustaining renal function. More recently, vitrification has also been demonstrated at liter scale and in porcine livers, although functional recovery and transplantation of cryopreserved human-sized organs have not yet been achieved. If successfully translated to porcine donor organs, cryopreservation could further decouple organ procurement from recipient surgery, enable long-term organ banking, facilitate donor–recipient matching, and substantially simplify geographical distribution. However, cryoprotectant toxicity, uniform cooling and rewarming, and translation to clinically sized organs remain major technical challenges [55, 56].

Digital technologies may further transform xenotransplantation logistics. Artificial intelligence could support donor herd management, organ allocation, transportation planning, and recipient matching. Real-time monitoring systems may integrate data from breeding facilities, perfusion devices, transportation networks, and transplant centers, creating highly coordinated and resilient supply chains.

If current progress continues, xenotransplantation may fundamentally reshape transplantation medicine. By replacing donor-dependent organ allocation with planned organ production and coordinated distribution networks, the field has the potential to transform transplantation from a scarcity-driven discipline into a predictable, scalable and safe therapeutic platform.
